# “Off-The-Shelf” allogeneic chimeric antigen receptor T-cell therapy for B-cell malignancies: current clinical evidence and challenges

**DOI:** 10.3389/fonc.2024.1433432

**Published:** 2024-07-09

**Authors:** Razan Mohty, Aleksandr Lazaryan

**Affiliations:** ^1^ Division of Hematology Oncology, Department of Medicine, O’Neal Comprehensive Cancer Center, University of Alabama at Birmingham Heersink School of Medicine, Birmingham, AL, United States; ^2^ Department of Blood and Marrow Transplantation and Cellular Immunotherapy, Moffitt Cancer Center, Tampa, FL, United States

**Keywords:** CAR (chimeric antigen receptor) T cells, allogeneic CAR T therapy, B-cell malignances, B-cell ALL, B-cell NHL, gene editing

## Abstract

Chimeric antigen receptor T-cell therapy (CAR T) has revolutionized the treatment landscape for hematologic malignancies, notably B-cell non-Hodgkin lymphoma (B-NHL) and B-cell acute lymphoblastic leukemia (B-ALL). While autologous CAR T products have shown remarkable efficacy, their complex logistics, lengthy manufacturing process, and high costs impede widespread accessibility and pose therapeutic challenge especially for patients in rapid need for therapy. “Off-the-shelf” allogeneic CAR T-cell therapy (alloCAR T) has emerged as a promising alternative therapy, albeit experimental to date. AlloCARTs are derived from healthy donors, manufactured by batches and stored, making them available off-the-shelf which lowers financial burden. Various gene editing techniques have been employed to mitigate graft-versus-host disease (GVHD) and host-versus-graft (HvG) to enhance alloCAR T persistence. In this review, we summarize available manufacturing techniques, current evidence, and discuss challenges faced with the use of alloCAR Ts.

## Introduction

Chimeric antigen receptor T-cell therapy (CAR T) has revolutionized the treatment of many hematologic malignancies, particularly B-cell non-Hodgkin lymphoma (B-NHL) and B-cell acute lymphoblastic leukemia (B-ALL). Four CAR T products have been FDA-approved for commercial use in patients with various B-NHLs and B-ALL and all of them are autologous as they are derived from the patient’s apheresed CD3+ T-cells. While currently approved CAR Ts have been very effective in potentially curing up to 35–40% of patients, many limitations are associated with their use. Due to their autologous nature, commercial CAR Ts require complex logistics and pose a significant regulatory burden on the treating institution and manufacturing companies. They require leukapheresis and manufacturing for each patient, similar to the production of individualized treatment. As a consequence of this manufacturing complexity, the cost of autologous CAR T production averages $373,000 to $475,000 per product (1). This, in turn, leads to increased costs for patients, the healthcare system, and society at large, eventually affecting access to this life-saving therapy for many patients.

A relatively long wait time has also been reported during the manufacturing of autologous CAR T-cells, with an average vein-to-vein time of 2–6 weeks, depending on the product used (1, 2). This may pose a therapeutic challenge in delivering CAR T-cell therapy to patients with rapidly progressive diseases who need timely treatment. Another limitation of using autologous CAR T cells is related to challenges in separating regular T-cells from circulating malignant cells during CAR T-cell manufacturing (3). This is particularly relevant for hematologic malignancies with a high likelihood of circulating disease and has led to CD19-negative selection during the manufacturing of brexucabtagene autoleucel (brexu-cel). Potential transduction of malignant cells was demonstrated by Ruella et al. in the case report of a patient with B-ALL treated with CD19-directed CAR T cells who relapsed 9 months later and was found to have CAR-transduced leukemic blasts (4). Other issues with autologous CAR T manufacturing are related to interpatient variability and often impaired quality and quantity of residual T-cells after prior line(s) of lymphotoxic therapy. Previous treatments and the tumor microenvironment have both been shown to affect T-cell fitness, thereby limiting CAR T-cell expansion *in vitro* and *in vivo* (5). This, in turn, contributes to the risk of manufacturing failure (5). Furthermore, given the correlation between CAR T-cell dose and response to treatment, any compromise to the number and quality of CAR T-cells in the final product may lead to inferior outcomes (6). Hence, healthy donor “off-the-shelf” allogeneic CAR T-cell therapy (alloCAR T) has emerged as a major alternative to overcome most of the aforementioned limitations (7). AlloCAR Ts can be derived from peripheral blood T-cells or NK cells of healthy donors, umbilical cord blood (UCB), or induced pluripotent stem cells (iPSCs) (7). Multiple batches of cryopreserved T-cells can be obtained from the same healthy donor, resulting in faster manufacturing of more accessible “off-the-shelf” CAR T products with potentially reduced financial burden to institutions and payers of this otherwise costly therapy. Furthermore, faster access to readily available alloCAR Ts allows easier re-dosing of the product if needed. This review summarizes current evidence for “off-the-shelf” alloCAR T therapy and discusses its therapeutic potential and challenges.

## Manufacturing of alloCAR Ts and gene editing techniques

**Figure 1:**
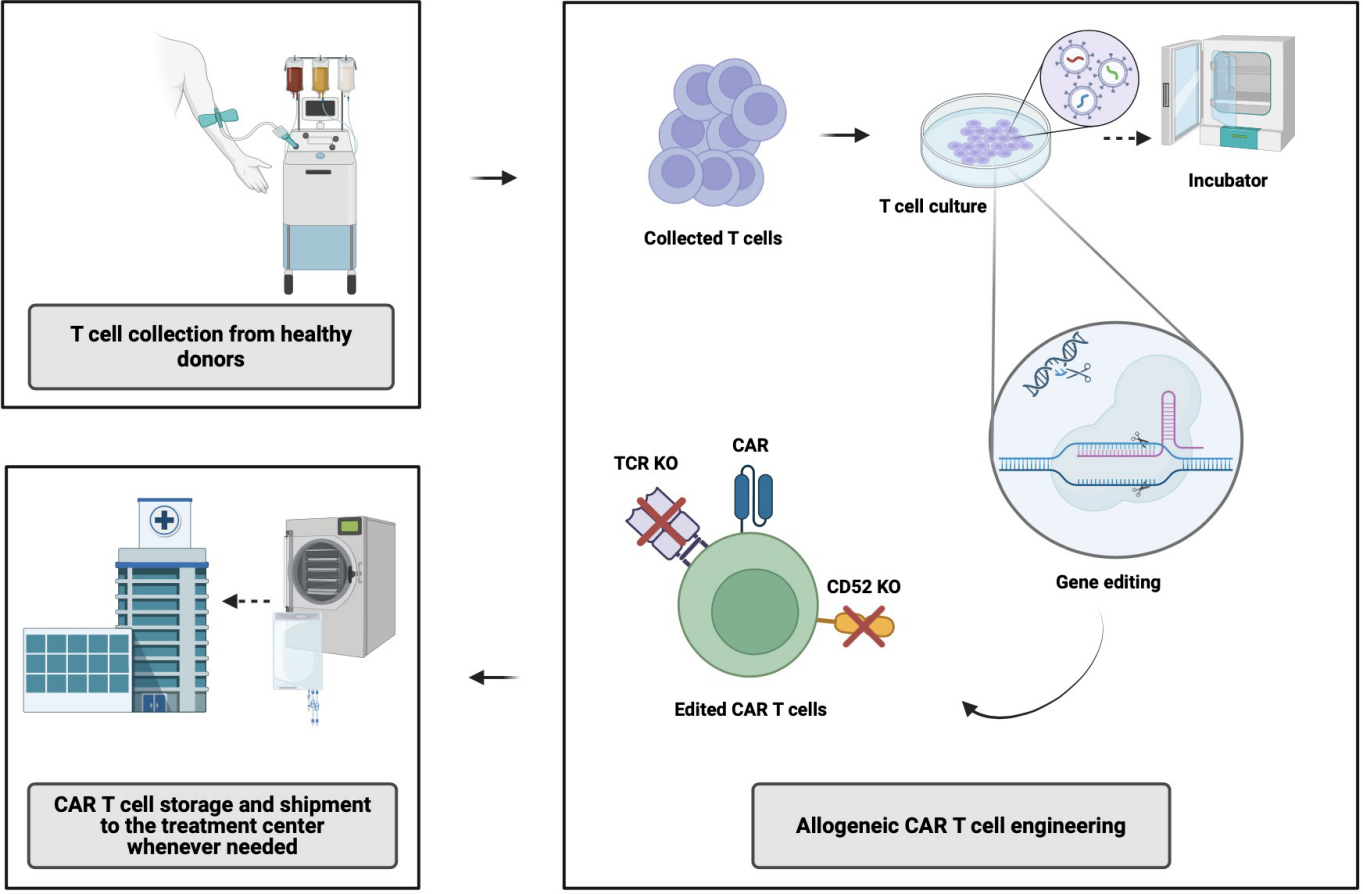
Allogeneic CAR T-cell manufacturing process. CAR, chimeric antigen receptor; TCR, T-cell receptor; KO, knock out. Created with BioRender.com.

AlloCAR T-cells from a single manufacturing batch have the potential to benefit multiple patients. The manufacturing of alloCAR Ts involves the use of immunologically intact and otherwise fit healthy-donor T-cells obtained via leukapheresis and subsequently cryopreserved (8). Various technologies have evolved around alloCAR T manufacturing, primarily focusing on immunologic incompatibilities between the recipient (patient) and healthy donor, with inherent risks of graft-versus-host disease (GVHD) and host-versus-graft (HvG), which can substantially limit the persistence and efficacy of alloCAR Ts. Generation of the CAR construct on the surface of alloCAR Ts involves established techniques such as viral vector-mediated transgenesis or gene knock-in editing for permanent insertion of recombinant DNA coding for a CAR and possibly additional genes (e.g., a suicide gene or a co-stimulatory receptor). This can be followed by the knockout of αβ T-cell receptor (*TCR*), β2-microglobulin (*β2M*), and *CD52* genes (8). The alloCAR Ts are subsequently cultured and expanded in the presence of cytokines. The remaining non-disrupted αβ TCR-positive cells are typically removed by negative selection using anti-αβ TCR antibodies. The final product is cryopreserved and shipped to the clinic for infusion whenever needed (**Figure 1**) (8).

Both gene- and non-gene editing technologies have been used to decrease the risk of GVHD as well as HvG, with the latter ultimately responsible for alloCAR T rejection following the immune reconstitution of the patient (7). Several methods have been developed to mitigate GVHD risk, including commonly targeted gene editing of the T-cell receptor constant α chain (*TRAC*) together with *β_2_M* gene knockout leading to disrupted α and/or β TCR and HLA MHC class I expression, respectively (8, 9). Other methodologies to mitigate those risks are based on the use of virus-specific memory T-cells, non-αβ T cells (e.g., γδ T cells), iPSCs, and donor-derived allogeneic T-cells in stem cell transplant recipients. Of note, knockout out of the HLA class I molecules could render CAR Ts more prone to be targeted by NK cells due to “missing self-signal” (8). Some studies have shown that knocking in HLA-E can prevent NK-cell-mediated elimination of those CAR Ts (8). Finally, the use of anti-CD52 monoclonal antibodies and *CD52* knockout within CAR Ts aim at decreasing the risk of HvG (10).

Several commonly used gene editing techniques include clustered regularly interspaced short palindromic repeats (CRISPR)/Cas systems, transcription activator-like effector nuclease (TALEN), zinc finger nuclease (ZFN), and ARCUS, among others (11–14) (Precision BioSciences I)^1^. These techniques share a common goal of creating specific DNA double-stranded breaks at pre-specified sites through chimeric nucleases. Gene repair mechanisms lead to gene inactivation (knock-out) or gene insertion (knock-in) through available exogenous DNA repair templates. They differ in their flexibility and efficacy in targeting the preselected DNA sites and the number of off-target cleavages.

## Clinical trials in B-NHL and B-ALL

### Allogeneic CAR Ts targeting CD19 antigen

CD19 is a transmembrane protein which is highly regulated and expressed throughout B-cell development (15). It is ubiquitously expressed across most B-cell malignancies, making it a successful target for many immunotherapies (15), including all approved autologous CAR T product. CD19 has also become the target of several alloCAR Ts which are currently under investigation (**Table 1**).

**Table 1:** Summary of major clinical trials utilizing allogeneic CAR T-cells in B-cell malignancies (final and/or interim data).

Ref/NCT	Trial phase	Disease	N	Manufacturing technique	Lympho depletion	Response	CRS	ICANS	GVHD	Other toxicity
CD19-directed CAR T cells										
ALPHA trial NCT03939026	Phase 1	LBCL FL	46	ALLO-501 *TRAC*, *CD52* loci editing, rituximab kill switch TALEN	FC, anti-CD52	ORR=75% CR=50%	Gr1–2 = 21.7% Gr3+=2%	None	None	Gr3+ infections=23.9%
ALPHA2 trial NCT04416984	Phase 1	LBCL	28	ALLO-501A *TRAC*, *CD52* loci editing TALEN	FC, anti-CD52	ORR=48% CR=28%	Gr1–2 = 11%	Gr1–2 = 21%	None	Cytopenia=57%
CARBON trial NCT04035434	Phase 1	LBCL	27	CTX110 CRISPR/Cas9	FC				None	
Shah et al. (16) NCT03666000	Phase 1/2a	B-NHL	13	PBCAR0191 *TRAC* locus editing ARCUS	FC	ORR=85% CR=62%	None	Gr3+=6%	None	Gr3+ neutropenia=15% Gr3+ infections=31%
		B-ALL	15		FC	CR/CRi=60%	Gr1–2 = 60% No Gr3+	Gr3+=6.7%	None	Gr3+ infections=40%
Shahid et al. (17) NCT01430390	Phase 1	B-NHL B-ALL	16	19–28 CAR EBV-CTLs	FC	NR	None	None	37%	NR
Xiao et al. (18) NCT05453669	Phase 1	B-NHL	15	RJMty19 Double-negative T- cells	FC	DL≥3: ORR=40%	Gr1–2 = 13% No Gr3+	None	None	None
Mehta et al. (19) NCT04629729	Phase 1	B-NHL B-ALL	12 (LBCL)	FT819–101 CRISPR/Cas *TRAC*/*TCR* editing 1XX signaling domain iPSC	FC	NR	Gr1–2 = 25% No Gr3+	None	None	None
ANTLER trial NCT04637763	Phase 1	B-NHL	5	cb-010: CRISPR *TRAC*/*PD-1* editing	FC	ORR=100% CR=80%	Grade 1 = 20%	Grade 3 = 20%	None	Gr3+ cytopenia and hypogamma globulinemia=50%
CALM trial NCT02746952	Phase 1	B-ALL	25	UCART19: TALEN *TRAC*/*CD52* editing	FC, anti-CD52	CR/CRi=100% MRD neg=48%	Gr1–2 = 56% Gr3+=24%	Gr1 = 24% Cr3+=4%	Gr1 = 8%	Gr4 cytopenia=16% Gr3 TLS=8%
CD20-directed CAR T cells										
Neelapu et al. (20) NCT04735471	Phase 1	B-NHL	9	ADI-001 γδ CAR T cells No gene editing	FC	ORR and CR=78%	Gr1 = 11%	Gr3 = 11%	None	NR
CD22-directed CAR T-cells										
BALLI-01 NCT04150497	Phase 1	B-ALL	18	UCART22 TALEN editing for *TRAC*/*CD52* editing	FC, anti-CD52	CR/CRi=33% MLFS=5.6%	Gr1–2 = 61%	None	Gr2 = 5%	Gr3+=72%
Dual directed CAR T cells										
NCT05105867	Phase 1	B-ALL	4	Truucar GC502 CD19/CD7 Technology NR *TRAC*/*CD7* editing	FC	CR/CRi=75%	Gr2 = 50% Gr3 = 50%	None	None	Gr3+ cytopenia=100%
Hu et al. (21) NCT05350787	Phase 2	B-ALL	7	ThisCART19A CD19 TCRαβ/CD3 No gene editing	FC, etoposide	CR/CRi=100% MRD neg=100%	Gr3+=25%	Gr3+=37.5%	NR	Fatal CRS/ infection=14%

Ref, reference; NCT, ClinicalTrials.gov ID; Nb of pts, Number of patients; CRS, cytokine release syndrome; ICANS, Immune effector cell-associated neurotoxicity syndrome; ORR, Overall response rate; CR, complete response; MRD, minimal residual disease; FC, fludarabine, cyclophosphamide; NHL, non-Hodgkin lymphoma; B-ALL, B-cell acute lymphoblastic lymphoma; LBCL, large B-cell lymphoma; FL, follicular lymphoma; Gr3+, ≥grade 3; NR, not reported; TRAC, T-cell receptor α constant; TLS, tumor lysis syndrome; TALEN, transcription activator-like effector nuclease; PD-1, programmed-cell death; DL, dose level; MLFS, morphologic leukemia free state; iPSC, induced pluripotent stem cell; EBV, Epstein Barr Virus; CTL, cytotoxic lymphocytes.

*Percentages were computed whenever only numeric data were published.

The ALPHA trial (NCT03939026), a phase 1, open-label, multicenter trial, evaluated the use of anti-CD19 alloCAR T (ALLO-501), which is TALEN gene-edited to disrupt *TRAC* and to knockout CD52 gene to decrease the risk of GVHD and GvH (22). The CD52 gene knock-out within alloCAR Ts also allowed the use of the anti-CD52 monoclonal antibody (ALLO-647) for better lymphodepletion along with standard fludarabine (30 mg/m^2^ per day) and cyclophosphamide (500 mg/m^2^ per day) given for 3 days. A total of 47 patients with large B-cell lymphoma (LBCL) and follicular lymphoma (FL) were enrolled, and 46 were treated (22). The study consisted of 2 cohorts: 39 patients received single infusion and 7 patients with at least stable disease at day 28 received another infusion of ALLO-647 and ALLO-501 (consolidation cohort). Twenty percent of the patients had received and failed prior autologous CAR T. No dose-limiting toxicity was reported. Only 1 patient developed grade ≥3 cytokine release syndrome (CRS), and no patient developed grade≥ 3 immune effector cell-associated neurotoxicity syndrome (ICANS). The objective response rate (ORR) and complete response (CR) rates were 75% and 50%, respectively, in the intention-to-treat population (22). The subsequent ALPHA2 trial enrolled patients with relapsed/refractory (R/R) LBCL who received a slightly modified product (aka ALLO-501A or cemacabtagene ansegedleucel [cema-cel]), which had the rituximab kill switch removed from ALLO-501 construct (23). In ALPHA2, CAR Ts were infused in a single (N=7) or repeated schedule (i.e., consolidation cohort, N=21) (24). No GVHD was reported. Only 3 and 6 patients developed low grade CRS and ICANS, respectively. Cytopenias were the most common adverse events in the entire cohort (57%). The ORR and CR rates were 48% and 28% among 28 evaluable patients, respectively. Longest CR durability was 15 months. After a median follow-up of 7.1 months from the single infusion in both ALPHA and ALPHA2 trials no dose-limiting toxicity (DLT), grade≥3 CRS/ICANS, or GVHD were reported in patients with LBCL treated with optimized lymphodepleting regimen. Among 12 evaluable patients, the ORR and CR rates were 66.7% and 58.3%, respectively, with a median duration of response of 23.1 months (25). Only five infusion reactions were reported following ALLO-647 (all grade 3) (15). Altogether, these data have supported the safety and efficacy of ALLO-501/ALLO-501A,however, longer follow-up is needed to evaluate the durability of these responses. The subsequent ALPHA3 trial will assess the use of cema-cel (ALLO-501A) as a consolidation therapy in patients with LBCL who have a minimal residual disease (MRD) detected after the first line of treatment. The MRD will be evaluated via investigational assay measuring circulating tumor (ct) DNA (PhasED-Seq™) (26).

The phase 1 Carbon trial evaluated CD19-directed alloCAR Ts (CTX110) in patients with R/R LBCL (27). CTX110 manufacturing involved the use of CRISPR/Cas-9 technology to disrupt *TRAC* and *β_2_M* genes (28). Patients who received prior autologous CAR Ts were excluded. The ORR and CR rates in patients who received dose level 3 (≥300 x 10^6^ CAR T cells; N=27) were 67% (18/27) and 47% (11/27), respectively. No GVHD was reported. The CRS and ICANS rates were 56% (18/32; all grade≤2) and 9.4% (3/32; 2 grade≥3), respectively. There were 7 serious adverse events attributed to CTX110, with 4 patients developing grade≥3 infections, including 1 fatal HHV6 infection (27). Despite its promising efficacy, CTX110 appeared to be associated with substantial toxicity. Thus, future studies will need to focus on preventing such alloCAR T-related toxicities by possibly incorporating prophylactic measures for patients at risk.

In the phase 1 ANTLER trial, the CD19-directed alloCAR T product (CB-010) was manufactured using CRISPR/Cas9 to knockout *TRAC* and *PD-1* genes to limit both GVHD and CAR T-cell exhaustion (29). Six patients with B-NHL were treated, and 5 were evaluable for response assessment. All patients responded, with 4 patients achieving CR. One patient developed grade 1 CRS together with grade 3 ICANS attributed to CB-010 but resolved within few days of therapy with tocilizumab and steroids. No GVHD was reported. This promising trial continues to accrue participants with the most recent updates by the manufacturer consistent with 94% ORR (15/16) and 69% CR (11/16) (30). While half of the 10 patients with R/R LBCL appeared to maintain CR at/or beyond 6 months, the final peer-reviewed and audited results of this trial are eagerly awaited to confirm these interim findings (NCT04637763).

Another innovative CD19-directed alloCAR T (PBCAR0191) was evaluated in a phase 1/2a trial for patients with B-cell malignancies (ie. B-NHL and B-ALL) (16). The PBCAR0191 CAR T manufacturing included a) single-step knock-in of the CD19 CAR into the *TRAC* locus via ARCUS gene editing; and b) more intense lymphodepletion (4 days of fludarabine 30 mg/m^2^/day and 3 days of cyclophosphamide 1000 mg/m^2^/day) to mitigate HvG. The ORR and CR rates in NHL cohort (N=16) were 85% (n=11/13) and 62% (n=8/13), respectively. Only 1 grade 3 ICANS (6%) and no severe CRS occurred. Five patients developed grade≥3 infections and 2 patients had severe neutropenia. The B-ALL cohort included 15 patients (31). The CR or CR with incomplete marrow recovery (CR/CRi) rate was 60% (9/15) with 4 patients bridged to allogeneic hematopoietic stem cell transplantation (alloHCT). Severe CRS and ICANS rates were similar to B-NHL patients. Six patients (%) developed severe infections. No GVHD was reported in any cohort (16, 31). While final trial results are still pending for both B-NHL and B-ALL cohorts, the available preliminary data appear promising with manageable safety and promising efficacy.

The CD19-directed UCART19 alloCAR T was designed by TALEN gene editing to disrupt *TRAC* and *CD52* (32). The UCART19 was evaluated in both pediatric (PALL trial) and adult (CALM trial) patients with B-ALL (32, 33). The combined data of PALL and CALM trials, including 7 children and 14 adult patients, demonstrated CR/CRi rate of 67% (14/21), with 4.1 months of median duration of response. Ten out 14 responders (71%) received subsequent alloHCT. CRS was reported in 91% (19/21) of patients, with only 3 (14%) having grade 3–4 CRS. Neurotoxicity was reported in 8 patients (38%). Six patients (32%) experienced grade 4 cytopenias. Two patients experienced treatment related death due to sepsis and CRS in 1 patient and pulmonary hemorrhage in the setting of prolonged cytopenia (33). The phase 1 CALM trial enrolled 25 patients (age range, 18–64 years). Eighteen patients (72%) received prior alloHCT, and 13 (52%) received cytoreductive therapy before lymphodepletion. Alemtuzumab was administered prior to UCART19, except for 3 patients due to concerns for increased risk of viral infections (32). Two patients developed grade 1 acute GVHD of the skin. CRS was reported in 20 patients (80%), of whom 6 had grade≥3 (24%) CRS. Seven patients (28%) had ICANS (only 1 grade 4) (32). Severe prolonged (day≥42) cytopenias occurred in 8 patients (32%). Moreover, severe (grade≥3) infections occurred in 7 patients including 2 fatal events among the alloHCT recipients. At a median follow-up of 12.8 months, all patients achieved CR/CRi, with 12 patients achieving MRD-negative remission. Of these patients, 9 (75%) received subsequent alloHCT at a median time of 1.7 months after UCART19. The median progression free survival (PFS) and overall survival (OS) were 2.1 and 13.4 months, respectively. Five patients received the second infusion of UCART19 for progressive disease or suboptimal response. All UCART19 batches were found to carry t (1, 14), which was expected following *TRAC* and *CD52* gene editing, however it was not associated with any lymphoproliferation. An allogeneic donor-derived product has been evaluated in 13 children with B-cell precursor ALL (34). The manufacturing of the products involved caspase-9 retrovirus or prodigy-lentivirus. All patients achieved CR MRD-negative in bone marrow at day 14 following alloCAR T infusion. Five of 6 patients with extramedullary involvement achieved CR at day 28Three patients underwent alloHCT. After 11 months of follow up, 8 patients maintained CR. Thus, the results of this trial appeared promising in the absence of increased toxicity or GVHD (34).

The anti-CD19-CD28-CAR, transduced into allogeneic Epstein-Barr Virus (EBV)-specific cytotoxic lymphocytes (19–28 CAR EBV-CTLs), had variable transduction efficiency with a median rate of 29% (range, 7–41%). A total of 16 patients with B-cell malignancies were treated. No CRS or ICANS were reported and 3 patients developed GVHD. Efficacy data were not reported to our knowledge. The OS was 81% at 12 months and 74% at 24 months (17).

The TCR-positive double-negative T-cells (DNT) targeting CD19 have an advantage of exploiting both natural and adaptive immunity including stem-cell-like memory T-cells (18). This DNT product (RJMty19) was evaluated in a phase 1 trial among patients with B-NHL and it was found to be overall safe with no reported grade≥3 toxicity. The ORR was 40% among the first 5 treated patients (18). Another phase 1 trial evaluated iPSC-derived CAR Ts (FT819) with a novel signaling domain (1XX) (19). It was hypothesized that this domain does not lead to CAR T exhaustion. The *TRAC* locus was edited via TCR knock-out. The precise technology used for gene editing was not reported (19), however FT819 was found to be safe based on the data from 12 patients with DLBCL (19).

Preclinical studies evaluating the use of CRISPR-edited CD19 CAR Ts (nU-CAR-T19) for B-cell malignancies showed promising efficacy *in vitro* and in animal models (9).

### Allogeneic CAR Ts targeting CD20 antigen

B-cell surface antigen CD20 was the first monoclonal antibody target leading to profound B-lymphocyte depletion. Surface expression of CD20 is present from early stages of B-cell differentiation (precursor B-cell) until the differentiated plasma cells. Hence, anti-CD20 monoclonal antibodies, such as prototypic rituximab, have been an integral part of B-NHL therapy for the past couple of decades (35). Cellular targeting of CD20 with ADI-001, a γδ alloCAR T has the advantage of not requiring any gene editing since γδ T-cells do not depend on MHC for their potent cytotoxicity (20). The safety and efficacy of ADI-001 were evaluated in a phase 1 trial of patients with R/R B-NHL. Eleven patients were enrolled, and 9 were evaluable. Two patients developed low grade CRS; 1 patient had grade 1 ICANS; and none had GVHD. The ORR and CR rates were 78% (7/9) (20). Studies evaluating the persistence of ADI-001 showed a median of 16,553 copies/µg at around 28 days from infusion (36). An ongoing trial is currently evaluating donor-derived CD20-directed alloCAR T therapy (LUCAR-20S) in R/R B-NHL (NCT04176913).

### Allogeneic CAR Ts targeting CD22 antigen

CD22 is another surface antigen expressed by all B-cells maturing from precursor B-cells until mature B-cells (35) and lost at the plasma cells stage. UCART22 is an alloCAR T directed against CD22, with *TRAC* and *CD52* disruption using TALEN gene editing (37). It was evaluated with or without the use of alemtuzumab in the BALL-01 phase 1 trial which enrolled 19 patients with R/R B-ALL (38) but only 8 were treated. Three patients developed CRS (all low grade), whereas no ICANS or GVHD were observed. Three patients achieved CRi at day 28, and 1 patient achieved a morphologic leukemia-free state (38).

### Dual-directed allogeneic CAR Ts

The advantages of dual targeting alloCAR Ts include overcoming single target antigen loss/downregulation, increasing tumor specificity and potentially limiting off-tumor effects via an inhibitory CAR (iCAR) design thereby leading to less toxicity (35). iCAR is a smart gated CAR T cell design that led to dual CAR inhibition via the “OR”, “NOT”, and “AND” logic gate.

TruUCAR GC502 is a CD19/CD7-directed alloCAR T with disrupted *TRAC* and *CD7* loci (to avoid fratricide) which was evaluated in R/R B-ALL (N=4) (39). All patients developed CRS (2 with grade 3 and 2 with grade 2). No ICANS or GVHD were reported. Three patients achieved CR/CRi, whereas the remaining patient achieved PR and subsequently underwent alloHCT on day 39 after alloCAR T (40). Another dual CD19/CD3 alloCAR T (ThisCART19A) was designed via non-gene-editing platform, based on intracellular retention of TCRαβ/CD3 complex. ThisCART19A was evaluated in 8 patients (7 evaluable) with B-ALL (21). Etoposide was added to standard lymphodepletion with fludarabine and cyclophosphamide. CR/CRi MRD negative rate was 100% with 4 patients remaining MRD negative state after the median follow up of 146 days. Severe CRS and ICANS were observed in 25% (2/8) and 37.5% (3/8) of patients, respectively. Another CD19/CD20 allogeneic bispecific CAR EBV CTLs were evaluated in preclinical studies demonstrating safety profile and potent antitumor activity (41). The CD20/CD22 alloCAR Ts with *TRAC* and *CD52* disrupted loci using TALEN technology were evaluated in a preclinical study showing robust activity *in vitro* and in animal models (42).

All major ongoing clinical trials evaluating healthy donor “off-the-shelf” alloCAR Ts in B-NHL and B-ALL are summarized in **Table 2**.

**Table 2:** Ongoing clinical trials utilizing allogeneic CAR T-cells in B-cell malignancies.

Study/NCT	Trial phase	Disease	Target	Product manufacturing	Technology	Status of the study
NCT05164042	Phase 1/2	B-ALL	CD19	NR	NR	UNK
NCT06014073	Phase 1/2	B-NHL	CD19	ATHENA CAR-T	CRISPR-Cas9 TRAC and Power3 genes knock out	Recruiting
NCT06256484	Phase 1	B-NHL	CD19	ATA3219	EBV T cells 1XX co-stimulatory domain	Recruiting
NCT04030195	Phase 1/2a	B-NHL CLL/SLL	CD20	PBCAR20A	ARCUS	Completed (not published)
NCT05106946	Phase 1	B-NHL	CD22	ThisCART22	Non-gene edited	Recruiting
NCT05691153	Phase 1	B-NHL	CD19/CD3	ThisCART19A CD19 TCRαβ/CD3	Non-gene edited	Recruiting
NCT06014762	Phase 1	B-cell malignancies	CD19/CD20	P-CD19CD20-ALLO1	Poseida Therapeutics	Recruiting
NCT05607420	Phase 1/2	B-NHL	CD20/CD22	UCART20x22	TALEN	Recruiting

B-ALL, B-cell Acute Lymphoblastic Leukemia; NR, not reported; UNK, unknown; B-NHL, B-cell non-Hodgkin lymphoma; CLL/SLL, chronic lymphocytic leukemia/small lymphocytic lymphoma; EBV, Epstein Barr virus.

## Limitations of alloCAR Ts and future perspectives

Despite multiple inherent advantages, promising efficacy, and manageable toxicity alloCAR Ts have certain limitations. Due to their allogeneic nature and expression of alpha beta TCR, the risk of GVHD presents a perpetual challenge together with the risk of HvG reaction jeopardizing persistence of alloCAR Ts and thereby their antitumor activity.

In the aforementioned clinical trials, the incidence of GVHD was low and largely limited to grades 1–2 as reported with UCART19 (32) and 19–28CAR EBV CTL (17), while most of other clinical trials did not report any GVHD. This highlights the effectiveness of technologies aiming at mitigating the risk of GVHD during manufacturing of allogeneic “off-the-shelf” CAR Ts. In contrast, the existing strategies to minimize HvG effect and thereby to enhance durability and efficacy of alloCAR Ts have been less successful. The available data on alloCAR T persistence are limited, given that most studies are still in their early stages. In the CALM trial, for example, UCART19 persisted in the blood for a median of 28 days (32). Only 5 patients had UCART19 persisting in the blood beyond day 42. Previous studies in autologous CAR Ts have shown a correlation between CAR T persistence and long-term response (43).

While in theory, GVHD and HvG can be mitigated or reduced through gene editing of *TRAC*, *CD52*, and *HLA-E* loci, together with the use of anti-CD52 monoclonal antibodies to deplete patient T-cells, this approach is contingent upon successful gene editing techniques. The use of anti-CD52 antibodies in various studies required *CD52* knock-out in alloCAR Ts. Although technologies used in gene editing have substantially improved, there is still a need to enhance the manufacturing platform to: a) improve the sensitivity and specificity of gene editing techniques in order to decrease off-target DNA editing; and b) mitigate the HvG effect through exploring novel allogeneic T-cell platforms with improved and more durable efficacy. Several ongoing studies are exploring innovative gene editing techniques focusing on increased specificity to target gene(s). As such, CRISPR/Cas9 technology has revolutionized the treatment of hematologic disease, while CRISPR/Cas12a system has further improved gene editing specificity without off-target effects. The CB-011, an alloCAR T, produced by CRISPR/Cas12a editing, is currently tested in patients with R/R multiple myeloma in the CaMMouflage trial (44). An innovative technology has been reported recently using transformer base editor (tBE), a multiplex editing that can target multiple genes simultaneously without reported off-target gene modification (45). Prime-Assisted Site-Specific Integrase Gene Editing (PASSIGE) is another novel technology demonstrating precision in multiplex editing without off-target effect (46). Upcoming trials will help to determine if this highly precise gene editing would translate into improved clinical outcomes. To mitigate CAR T exhaustion, a novel platform has been investigated in autologous CAR Ts for lymphoma, involving γδ TCR coupled with a costimulatory agent as an alternative to CAR (47). While this approach has not yet been explored in allogeneic T-cell therapies, it could potentially reduce GVHD risk and increase effectiveness of alloCAR Ts.

Secondary hematologic malignancies such as T-cell lymphoproliferations, have been reported among 22 autologous CAR T recipients by the end of 2023, leading to addition of the boxed warning by the FDA to CAR T-cell therapies. In theory, similar risk may exist with alloCAR Ts produced by gene editing techniques and/or the use of viral vector platforms. In fact, secondary hematologic malignancies have been previously reported following CRISPR edited gene therapy for sickle cell disease due to vector mediated oncogenesis. It remains unclear whether HvG effect observed with alloCAR Ts would also limit rare instances of secondary lymphoproliferation due to unintentional transduction of healthy-donor T-cells. On another hand, the use of novel non-gene editing alloCAR T platforms (e.g. ThisCART) may further reduce the risk of any secondary oncogenesis.

In conclusion, “off-the-shelf” alloCAR Ts have emerged as a promising therapeutic avenue for patients with B-cell malignancies. Early reports have demonstrated robust efficacy and limited toxicity of alloCAR Ts, however their clinical superiority in comparison to autologous CAR Ts remains uncertain. Ongoing studies harnessing novel technologies in manufacturing next generation alloCAR Ts hold promise in limiting further toxicities while improving persistence and efficacy of alloCAR Ts.

## Author contributions

RM: Conceptualization, Writing – original draft, Writing – review & editing. AL: Conceptualization, Supervision, Writing – original draft, Writing – review & editing.

## Footnotes

1 Precision BioSciences I. A precise platform for clinical gene editing. Biopharma Dealmakers (Biopharm Deal). Available at: https://www.nature.com/articles/d43747-022-00219-x.
